# Wearable Biosensors for Continuous Monitoring of Chronic Kidney Disease: Materials, Biofluids, and Digital Health Integration

**DOI:** 10.3390/bios16050287

**Published:** 2026-05-15

**Authors:** Anupamaa Sivasubramanian, Shankara Narayanan, Gymama Slaughter

**Affiliations:** 1 Center for Bioelectronics, Old Dominion University, Norfolk, VA 23508, USA; 2 Andhra Pradesh MedTech Zone (AMTZ), Visakhapatnam 530031, AP, India; 3 Accurex Biomedical Pvt. Ltd., Visakhapatnam 530031, AP, India; 4 Department of Electrical and Computer Engineering, Old Dominion University, Norfolk, VA 23508, USA

**Keywords:** chronic kidney disease, wearable biosensors, electrochemical sensors, point-of-care diagnostics, sweat and interstitial fluid monitoring

## Abstract

Chronic kidney disease (CKD) is a progressive and irreversible disorder affecting over 850 million individuals globally and is associated with significant morbidity, mortality, and healthcare burden. Conventional diagnostic approaches rely on intermittent laboratory measurements, including serum creatinine, estimated glomerular filtration rate (eGFR), and urinary albumin, which provide limited temporal resolution and fail to capture dynamic physiological changes. Recent advances in wearable biosensing technologies offer new opportunities for continuous, non-invasive monitoring of biochemical and physiological markers relevant to renal function. This review provides a comprehensive analysis of wearable biosensors for CKD monitoring, focusing on sensing mechanisms (electrochemical, optical, and field-effect transistor), biofluid interfaces (sweat, interstitial fluid, and saliva), and materials engineering strategies enabling flexible, high-performance devices. Emphasis is placed on biofluid transport dynamics, analytical performance across sampling matrices, and system-level integration with wireless communication and digital health platforms. Key challenges limiting clinical translation, including biofouling, enzymatic instability, and variability in biofluid composition, are examined—alongside emerging solutions such as antifouling interfaces, synthetic recognition elements, and multimodal sensing architectures. Finally, regulatory pathways and the role of artificial intelligence in digital nephrology are discussed. This review highlights the potential of wearable biosensors to transform CKD management through continuous monitoring, early detection, and personalized therapeutic intervention.

## 1. Introduction

Chronic kidney disease (CKD) is a major global health challenge affecting approximately 850 million individuals worldwide and is projected to become a leading cause of mortality by 2040 [1]. The disease is characterized by progressive nephron loss, metabolic dysregulation, and accumulation of uremic toxins, ultimately leading to end-stage renal disease (ESRD) requiring dialysis or transplantation [1,2,3,4]. Early-stage CKD is often asymptomatic, contributing to delayed diagnosis and poor clinical outcomes. Current clinical assessment relies on biomarkers such as serum creatinine, estimated glomerular filtration rate (eGFR), blood urea nitrogen, and urinary albumin [4,5,6]. However, these measurements provide only episodic assessments of renal function and are influenced by physiological variability, including muscle mass, hydration status, and diet. Consequently, they do not capture dynamic biochemical changes or enable early detection of disease progression [6,7,8,9,10,11]. At the same time, the accuracy of key biomarkers remains constrained by methodological limitations. Serum creatinine measurement, commonly performed using the Jaffe reaction, is susceptible to interference from non-creatinine chromogens, leading to potential overestimation [12]. Such analytical variability further limits the reliability of creatinine-based kidney function assessment in clinical decision-making.

In addition, impaired sodium excretion and neurohormonal activation promote extracellular fluid expansion, contributing to hypertension, cardiovascular remodeling, and heart failure in CKD patients [13]. These limitations highlight the need for wearable biosensors as a promising approach for continuous, minimally invasive monitoring of CKD-related biomarkers. These systems integrate biorecognition elements with miniaturized transducers, flexible substrates, and wireless communication to enable real-time detection of analytes in peripheral biofluids such as sweat, interstitial fluid (ISF), and saliva [14,15,16,17,18,19,20]. Unlike conventional diagnostics, wearable platforms support longitudinal tracking and remote patient management, forming a foundation for emerging digital health frameworks.

Electrochemical, optical, and field-effect transistor (FET) sensing mechanisms have been adapted for wearable formats capable of detecting metabolites and electrolytes relevant to CKD, including urea, creatinine, uric acid, albumin, sodium, and potassium [16,21,22,23,24,25,26,27,28,29]. Compared with conventional diagnostics, wearable biosensors enable continuous data acquisition, supporting longitudinal biomarker tracking and earlier detection of electrolyte instability and CKD progression. However, translation of wearable biosensors to CKD monitoring presents significant challenges. Unlike diseases characterized by a single dominant biomarker, CKD is a complex systemic disorder involving multiple interacting metabolic and physiological processes. Effective monitoring therefore requires detection of multiple analytes and physiological parameters rather than reliance on a single molecular marker.

While recent studies have examined wearable biosensors for general healthcare diagnosis [14,15,16,17,18,19,20], focused analysis of CKD-specific biomarkers and biofluid transport dynamics remains limited. In particular, the integration of renal pathophysiology, biofluid transport mechanisms, and wearable sensing technologies has not been addressed. This review provides a unified framework that integrates (i) CKD pathophysiology and clinically relevant biomarkers, (ii) biofluid transport dynamics governing analyte availability in peripheral fluids such as sweat and interstitial fluid, and (iii) the performance characteristics and limitations of wearable sensing modalities under real-world conditions. By explicitly linking physiological transport with sensor response across biofluids, this work highlights critical constraints often overlooked in existing reviews, including temporal delays, concentration variability, and correlation with blood-based diagnostics. In addition, comparative analysis of sensing strategies, biomarker suitability for continuous monitoring, and system-level integration with digital health platforms and artificial intelligence are presented, providing a bridge between laboratory-scale sensor development and clinically deployable wearable systems for CKD management.

## 2. Wearable Biosensing: Biomarkers, Biofluids, and Sensing Modalities

Wearable biosensors are integrated analytical systems designed to continuously monitor biochemical and physiological parameters in a non-invasive or minimally invasive manner. Advances in flexible electronics, microfabrication, and biointerface engineering have enabled compact devices capable of detecting metabolites, electrolytes, and physiological signals in real time. A comparison of key CKD biomarkers and their relevance to wearable sensing is summarized in Table 1. A typical wearable biosensor comprises a biorecognition element for selective target interaction, a transducer that converts this interaction into a measurable signal, signal conditioning and processing electronics, a power source, and wireless communication modules for data transmission [15,16,19,20]. These components collectively define sensor architecture; however, overall system performance is ultimately governed by the choice of transduction mechanism and the ability to maintain stable operation in complex biological environments.

Additionally, wearable biosensors primarily rely on peripheral biofluids such as sweat and ISF, with blood serving as the clinical reference standard. However, analyte transport from blood to these compartments occurs via diffusion, introducing temporal delays and variability in concentration profiles. This physiological decoupling between central and peripheral compartments represents a fundamental limitation, as biomarker levels measured in wearable-accessible fluids may not directly reflect clinically relevant blood concentrations. In addition, variability in sweat rate, skin permeability, and environmental conditions further complicates quantitative interpretation.

While advances in electrochemical, optical, and FET-based sensing have improved analytical sensitivity, the primary challenge in wearable CKD monitoring is not solely detection capability but the reliable correlation and interpretation of biomarker signals across biofluids. Consequently, accurate calibration strategies, multimodal sensing integration, and data-driven modeling approaches are required to bridge the gap between peripheral measurements and clinically actionable insights.

**Figure 1:**
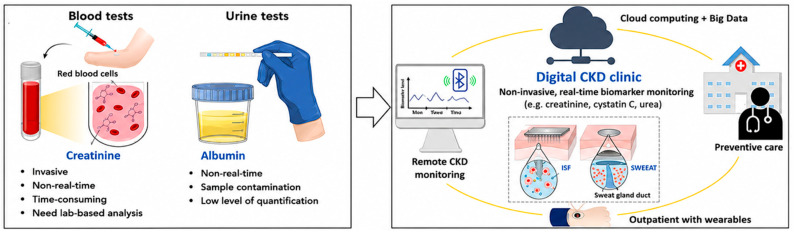
Wearable digital healthcare concept for CKD monitoring [33].

Accordingly, multiple sensing strategies, including electrochemical, optical, FET, and impedance-based approaches, have been explored for CKD biomarker monitoring [21,23,26,32,33,34,35,39,40,41,42,43]. The integration of these sensing modalities with digital health infrastructure, including wireless data transmission and AI-based analytics, enables multimodal data fusion and supports real-time monitoring and personalized disease management (Figure 1). However, the successful clinical translation of these systems will depend on overcoming persistent challenges related to biofluid variability, signal calibration, and long-term reliability.

### 2.1. Electrochemical Biosensors

Electrochemical biosensors are the most widely investigated wearable platforms due to their high sensitivity, low power requirements, and compatibility with miniaturized electronics [15,23]. These sensors detect analytes by measuring electrical signals such as current, voltage, or impedance generated from biochemical reactions at the electrode interface. In wearable systems, detection is typically implemented using amperometric, potentiometric, or impedance-based methods. Amperometric sensors commonly rely on enzymatic reactions; for example, urease-catalyzed hydrolysis enables electrochemical detection of urea via ammonium ion generation [32,33]. Potentiometric sensors employ ion-selective membranes incorporating conductive polymers such as PEDOT or poly(3-octylthiophene-2,5-diyl) for selective detection of sodium and potassium ions [36].

Representative wearable platforms include flexible sweat-based patches with urease-functionalized electrodes for continuous urea monitoring and ion-selective sensors for real-time electrolyte detection in epidermal formats. These systems demonstrate micromolar sensitivity and strong compatibility with wearable integration, underscoring the relative maturity of electrochemical sensing. However, their long-term performance is limited by biofouling, enzyme degradation, and signal drift, which can compromise measurement stability during continuous operation. These challenges necessitate frequent calibration and restrict their reliability for prolonged, real-world monitoring, thereby limiting their effectiveness as standalone sensing modalities.

### 2.2. Optical and Colorimetric Sensors

Optical biosensors detect biomarkers by converting biochemical interactions into measurable signals such as absorbance, fluorescence, or color change. These systems offer advantages including high selectivity, multiplexing capability, and electrical isolation. Colorimetric platforms are particularly suitable for wearable applications due to their compatibility with simple readout systems such as smartphone cameras [34,35]. Enzyme-mediated colorimetric reactions have been explored for metabolites, including urea [35].

Wearable implementations include microfluidic colorimetric patches and fluorescence-based systems for sweat biomarker analysis, with smartphone-assisted platforms enabling low-cost, semi-quantitative monitoring. However, their application in CKD monitoring is constrained by sensitivity to environmental and physiological variability, including ambient light interference, photobleaching, and fluctuations in optical path length due to changes in sample thickness. These factors introduce significant variability in signal intensity, limiting quantitative accuracy and reproducibility in real-world conditions, and thereby restricting their reliability as standalone sensing modalities.

### 2.3. Field-Effect Transistor Biosensors

FET-based biosensors detect analytes through modulation of semiconductor channel conductivity induced by molecular binding at the sensor surface. Binding of charged biomolecules alters the local electric field, resulting in measurable conductivity changes. Materials such as graphene, silicon nanowires, and two-dimensional semiconductors are widely used due to their high surface-to-volume ratios and favorable electronic properties, enabling rapid, label-free detection [39,40]. Wearable FET-based sensors have demonstrated high sensitivity for biomarkers such as creatinine and uric acid using nanomaterial channels. However, despite their low theoretical detection limits and rapid response, their performance in physiological fluids is fundamentally constrained by Debye screening. This further attenuates electric field interactions in high ionic strength environments such as sweat and interstitial fluid. This limitation restricts effective sensing to within the Debye length, thereby reducing practical sensitivity and limiting their standalone applicability in real-world wearable systems.

### 2.4. Bioimpedance and Physiological Sensors

Physiological monitoring is an important component of CKD management. Bioimpedance spectroscopy provides a noninvasive method for assessing fluid status and body composition by measuring tissue impedance across multiple frequencies [41,42,43]. Given that CKD is frequently associated with fluid retention and extracellular volume expansion, impedance-based measurements offer valuable insight into hydration status and cardiovascular risk. Wearable bioimpedance systems have been deployed in both clinical and ambulatory settings to track extracellular fluid changes, including dialysis-related fluid shifts. While these systems correlate well with clinical indicators of fluid imbalance and provide complementary physiological context, they do not directly measure biochemical biomarkers. This limitation constrains their standalone diagnostic utility and underscores the need for integration with biochemical sensing modalities to enable clinically actionable CKD monitoring.

### 2.5. Multimodal Sensing Systems

Multimodal wearable sensing systems have emerged as a promising approach for comprehensive CKD monitoring, reflecting the inherently complex and multifactorial nature of CKD pathophysiology. By integrating biochemical sensing with complementary physiological modalities, such as temperature, sweat rate, and bioimpedance, these platforms enable cross-validation of biomarker signals while providing essential physiological context for data interpretation. Recent advances in flexible electronics, microfluidic sampling, and wireless communication have accelerated the development of compact epidermal and textile-based systems capable of simultaneous multi-parameter monitoring [14,16,44,45,46,47,48].

Despite this progress, significant technical and translational challenges remain. A comparative overview of sensing modalities is provided in Table 2. Electrochemical sensors, while the most mature modality due to their high sensitivity, low power consumption, and compatibility with wearable formats, continue to face limitations related to biofouling, enzyme instability, and long-term signal drift under continuous operation. Optical and colorimetric approaches offer advantages in multiplexing and electrical isolation but are inherently susceptible to environmental variability, including ambient light interference and changes in optical path length, which can compromise measurement reliability outside controlled settings. FET-based sensors provide rapid, label-free detection with high theoretical sensitivity; however, their performance in physiological fluids is fundamentally constrained by Debye screening effects in high ionic strength environments, limiting their practical utility for real-world applications.

Furthermore, physiological sensing modalities such as bioimpedance provide valuable information regarding fluid status and cardiovascular risk; however, they lack direct biochemical specificity and therefore cannot independently capture disease-relevant molecular changes. As a result, the integration of biochemical and physiological sensing modalities represents a necessary, yet not fully optimized, strategy for comprehensive CKD monitoring. Critically, current multimodal systems often demonstrate proof-of-concept functionality but fall short of achieving robust, long-term operation in complex biological environments. Challenges related to sensor calibration, inter-signal interference, data fusion, and clinical validation remain insufficiently addressed. In particular, the lack of standardized frameworks for multimodal data integration and interpretation limits the ability to translate sensor outputs into clinically actionable insights. While multimodal platforms represent the most promising pathway toward CKD monitoring, their successful translation will depend on overcoming persistent limitations in stability, reliability, and real-world validation.

### 2.6. Representative Wearable CKD Biosensor Systems

Sweat-based epidermal patches incorporating microfluidic sampling and electrochemical sensing have been widely explored for continuous monitoring of urea, sodium, potassium, and uric acid during physiological activity [18,22]. Additionally, minimally invasive microneedle-based sensors enable access to ISF, offering improved correlation with blood biomarkers and facilitating detection of metabolites such as glucose, urea, and electrolytes. However, diffusion-driven transport between blood and peripheral compartments introduces temporal delays relative to plasma concentrations, typically on the order of minutes [30,31], which can complicate real-time interpretation of biomarker dynamics.

Moreover, multimodal systems integrating biochemical sensing with physiological measurements such as temperature or bioimpedance have been developed to improve contextual interpretation under dynamic conditions [14,16]. Despite these advances, most wearable CKD biosensor systems remain at the prototype stage, with limited large-scale clinical validation and insufficient long-term performance data. This lack of validation, combined with variability in biofluid composition and temporal lag effects, constrains the reliability and clinical translatability of current platforms. Consequently, a qualitative comparison of sensing metrics across biofluids and modalities is essential for identifying viable pathways toward robust deployment, as summarized in Table 3.

## 3. Wearable CKD Biosensors by Biofluid

**Figure 2:**
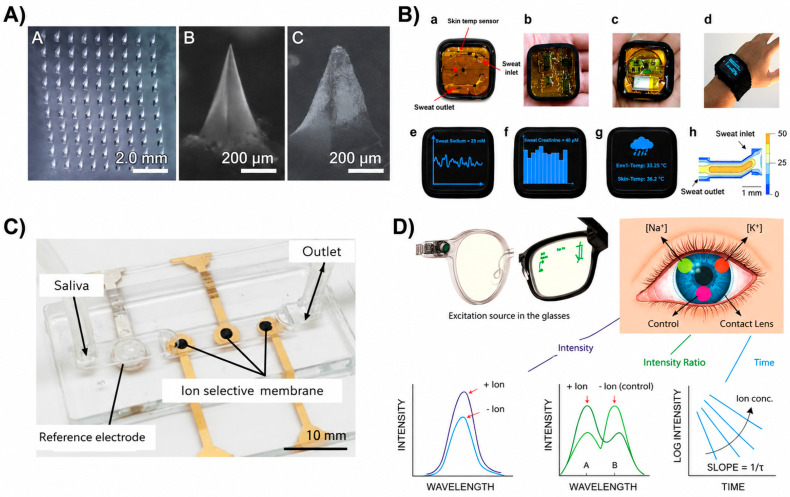
Wearable biosensors across biofluids. (**A**) Microneedle ISF sensor (A) PVA-based hydrogel MN patch containing a 10 × 10 microneedle array, (B) the dry-state morphology prior to skin insertion and (C) the swollen hydrated structure following 12 h insertion into ex vivo porcine skin, demonstrating fluid uptake and hydrogel expansion [30], (**B**) sweat patch (a) microfluidic sweat inlet/outlet integrated with a thermistor; (b) environmental temperature sensing module; (c) wireless battery-powered system; (d) on-body wearable sensor configuration; (e) sweat sodium readout; (f) sweat creatinine readout; (g) environmental and skin temperature monitoring interface; and (h) simulated flow profile within the potentiometric microfluidic channel [53], (**C**) saliva sensor [37], and (**D**) smart contact lens for tear analysis [69], illustrating diverse platforms for continuous, noninvasive biochemical monitoring.

The physiological characteristics and transport dynamics of biofluids critically influence the design and performance of wearable biosensors. Accordingly, wearable CKD sensing platforms are often categorized based on the biofluid used for biomarker sampling (Figure 2). Each biofluid presents inherent trade-offs in terms of accessibility, correlation with blood biomarkers, and compatibility with wearable technologies. Among these, ISF (Figure 2A) and sweat (Figure 2B) have received the most attention due to their suitability for minimally invasive sampling via epidermal and microneedle-based systems [15,18,22,30,31,48,67,68,69]. In contrast, sweat provides a highly accessible sampling medium but exhibits significant variability in composition due to factors such as sweat rate, environmental conditions, and individual physiology, complicating quantitative interpretation.

Other biofluids, including saliva (Figure 2C) and tears (Figure 2D), have also been explored but remain less established for CKD monitoring due to lower biomarker concentrations and limited clinical validation [37,38,69,70]. Collectively, these biofluid-specific limitations highlight a fundamental challenge in wearable biosensing: biomarker concentrations measured in peripheral compartments do not consistently reflect systemic physiological states, thereby constraining the reliability and clinical translatability of wearable platforms.

### 3.1. Sweat

Flexible epidermal patches integrated with microfluidic interfaces have been developed for detecting electrolytes and metabolites such as sodium, potassium, urea, and uric acid using electrochemical and optical methods [25,30,31,37,38,68]. The noninvasive and continuous accessibility of sweat makes it highly attractive for wearable applications. However, its clinical utility is fundamentally constrained by significant physiological variability. Sweat composition is strongly influenced by sweat rate, temperature, hydration status, and inter-individual differences, leading to inconsistent biomarker concentrations. Furthermore, correlation with blood biomarkers is often weak or analyte-dependent, particularly for urea and creatinine, limiting its reliability as a quantitative surrogate. These factors introduce substantial uncertainty in data interpretation and necessitate robust calibration and normalization strategies. Therefore, sweat-based sensing is generally better suited for monitoring temporal trends rather than providing absolute biomarker concentrations.

### 3.2. Interstitial Fluid (ISF)

Microneedle-based sensors enable minimally invasive access to ISF, allowing sampling of metabolites such as glucose, electrolytes, urea, and creatinine with improved physiological relevance [30,31]. This closer correlation with blood biomarkers enhances the potential for clinically meaningful measurements compared to other peripheral biofluids. However, ISF-based sensing remains limited by transport-related delays. Diffusion of analytes from the vascular compartment into ISF introduces temporal lag, typically on the order of minutes, which can compromise the accuracy of real-time monitoring under dynamic physiological conditions. This lag creates a mismatch between measured and actual systemic biomarker levels, particularly during rapid physiological changes, thereby complicating clinical interpretation. In addition, microneedle-based systems present challenges related to user comfort, long-term wearability, and biofouling, which can impact signal stability over extended use. These limitations constrain the reliability of ISF sensing as a standalone modality and highlight the need for calibration strategies, temporal compensation models, and integration with complementary sensing approaches to enable robust, continuous CKD monitoring in real-world settings [30,31].

### 3.3. Saliva and Tear Fluid

Saliva and tear fluid offer fully noninvasive sampling routes and have been explored for wearable sensing using platforms such as mouthguard sensors and smart contact lenses depicted in Figure 2C,D [37,38,69,70]. These biofluids contain metabolites and electrolytes derived from plasma through diffusion and glandular processes. However, their applicability to CKD monitoring remains limited. Correlation with systemic renal biomarkers is generally weaker compared to sweat and ISF, reflecting differences in transport mechanisms, dilution effects, and local physiological regulation. As a result, biomarker concentrations in saliva and tears may not reliably represent systemic renal function, reducing their diagnostic accuracy for CKD monitoring. While these biofluids offer advantages in accessibility and user comfort, their limited correlation with clinically relevant biomarkers constrains their utility for quantitative assessment. Further, their role in CKD monitoring remains restricted, and they are more likely to serve as complementary sensing routes rather than primary diagnostic modalities in wearable systems.

### 3.4. Transport and Interface Considerations

Regardless of the biofluid, sensor performance is fundamentally governed by mass transport processes at the biointerface. Analytes must diffuse from physiological compartments such as blood or ISF through biological barriers before reaching the sensing surface. These processes depend on concentration gradients, diffusion coefficients, and local fluid dynamics. Transport limitations can introduce delays between physiological changes and sensor response, particularly in ISF-based systems, where diffusion-driven lag can decouple measured signals from real-time systemic conditions. In sweat-based sensing, variations in sweat rate and fluid turnover can alter analyte concentrations near the sensor interface, contributing to significant signal variability. In addition, biofouling and protein adsorption can impede analyte transport and degrade sensor performance over time, leading to signal drift and reduced stability [57].

These coupled transport and interfacial effects limit the temporal accuracy, quantitative reliability, and long-term stability of wearable biosensors, thereby constraining their ability to provide clinically actionable measurements. Understanding and mitigating these phenomena through improved sensor design, calibration strategies, and data-driven correction models is therefore essential for the development of reliable wearable CKD biosensing platforms.

### 3.5. Comparative Perspective Across Biofluids

Each biofluid presents distinct advantages and limitations for CKD monitoring. Blood remains the clinical gold standard due to its direct representation of systemic physiology; however, its invasive nature limits its suitability for continuous monitoring. ISF provides a favorable compromise, offering improved correlation with blood biomarkers while enabling minimally invasive sampling, although transport-related temporal delays must be considered [30,71]. Sweat is the most accessible biofluid for continuous wearable sensing; however, its high variability and weaker correlation with blood biomarkers limit its utility for precise quantitative analysis. Saliva and tear fluid offer convenient, fully noninvasive sampling routes but currently lack sufficient clinical relevance and validation for CKD diagnostics. These trade-offs highlight a fundamental limitation in wearable CKD biosensing: no single biofluid simultaneously provides high accessibility, strong correlation with systemic biomarkers, and reliable quantitative accuracy. As a result, effective CKD monitoring needs integrated approaches that combine multiple biofluids, sensing modalities, and data-driven correction strategies to achieve clinically actionable measurements. A comparative summary of key biofluids for wearable CKD monitoring is provided in Table 4.

ISF-based sensing, despite its relatively short lag (~5–15 min), can still provide clinically useful insights for tracking trends and enabling early warning systems, particularly when combined with predictive modeling approaches. In contrast, sweat-based sensing faces greater limitations due to longer and more variable delays (~10–30 min), as well as weaker correlation with blood biomarkers, reducing its reliability for precise clinical decision-making. Therefore, sweat-based platforms are better suited for monitoring temporal trends rather than providing accurate real-time measurements. While temporal delays in ISF and sweat are generally acceptable for routine physiological monitoring, their clinical significance increases in the context of CKD. Although CKD typically progresses gradually, acute events such as sudden electrolyte imbalances (e.g., hyperkalemia) or rapid changes in uremic toxin levels require near real-time detection. In such scenarios, even short delays can lead to discrepancies between measured and actual physiological states.

Moreover, these transport-related delays highlight a fundamental limitation of wearable biosensing: the inability to fully capture rapid physiological changes in real time. As a result, current wearable systems are unlikely to function as standalone diagnostic tools for CKD and instead must be integrated with established clinical testing frameworks, supported by calibration strategies and data-driven correction models to improve interpretability and clinical utility.

### 3.6. Prioritization of Biomarkers for Wearable CKD Monitoring

**Figure 3:**
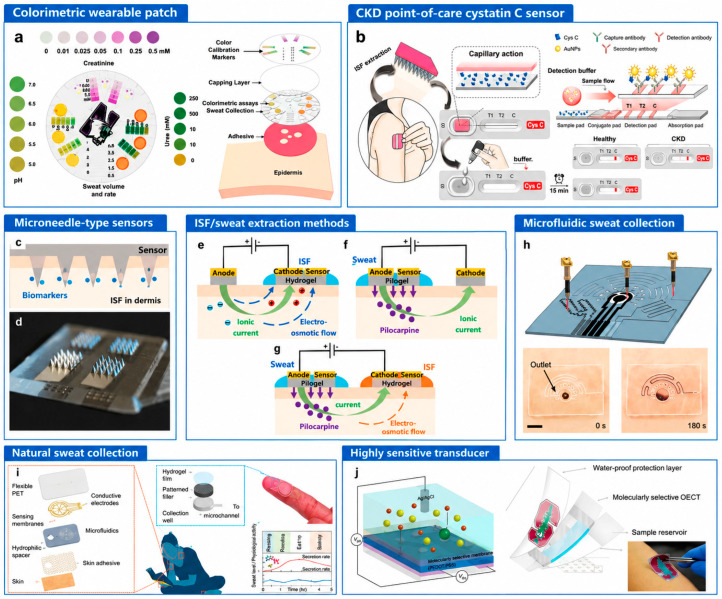
Representative wearable and minimally invasive biosensing platforms for CKD monitoring across multiple biofluids, including (**a**) colorimetric wearable patch (creatinine and urea), (**b**) ISF-based cystatin C sensor, (**c**,**d**) microneedle-type sensor, (**e**,**f**) ISF and sweat extraction methods (**g**) wearable patch, (**h**) laser-engraved microfluidic channels and (**i**) microfluidic channel for sweat collection, (**j**) OECT transducer [33].

CKD monitoring involves multiple biomarkers; however, their suitability for wearable applications is governed not only by clinical relevance but also by detectability in peripheral biofluids, compatibility with sensing technologies, and stability under continuous monitoring conditions. To address this, a structured prioritization framework has been introduced (Figure 3), categorizing biomarkers based on their practical feasibility for wearable deployment.

Figure 3 illustrates a diverse range of wearable and minimally invasive biosensing technologies developed for CKD monitoring, spanning sweat-based patches, microneedle-based ISF sensors, saliva and tear-based platforms, and integrated microfluidic and electrochemical systems. These devices demonstrate significant progress toward continuous, real-time monitoring through flexible electronics, wireless communication, and smartphone integration. Despite this technological diversity, most platforms remain at the prototype stage and face common limitations related to biofluid variability, transport-induced delays, sensor stability, and limited clinical validation. The primary challenge is not the availability of sensing technologies, but the reliable translation of measured signals into clinically meaningful information.

## 4. Materials and Device Engineering for Wearable CKD Biosensors

Material selection influences key performance parameters, including sensitivity, selectivity, mechanical durability, and operational stability in complex biological environments. Progress in nanostructured electrodes, conductive polymers, flexible substrates, and engineered biointerfaces has significantly enhanced the analytical performance of wearable biosensors [72,73]. These materials improve charge transfer efficiency, facilitate biomolecule immobilization, and enable conformal contact with soft, dynamic biological tissues. In addition to improving signal transduction, advanced materials support scalable fabrication strategies compatible with flexible electronics and microfabrication processes. Nanostructured carbon materials, metal nanostructures, conductive polymer composites, and antifouling coatings have therefore become core components of wearable sensing systems targeting renal biomarkers such as urea, creatinine, and electrolytes in peripheral biofluids.

However, despite these advances, material innovations alone do not fully address the fundamental challenges associated with wearable biosensing, including biofluid variability, transport-induced delays, and long-term signal drift. While antifouling coatings and engineered interfaces can mitigate surface degradation, biofouling and interfacial instability remain significant barriers to prolonged operation. Moreover, improvements in sensitivity and selectivity do not necessarily translate to improved clinical accuracy when biomarker concentrations in peripheral biofluids are weakly correlated with systemic physiological states. As a result, material engineering impact is ultimately constrained by broader system-level limitations, highlighting the need for integrated approaches that combine advanced materials with robust calibration strategies, multimodal sensing, and data-driven interpretation frameworks.

### 4.1. Nanostructured Electrode Materials

Nanostructured electrode materials are widely used in wearable biosensors due to their high surface-to-volume ratios and tunable electronic properties, which enhance electrochemical signal transduction. Carbon-based nanomaterials, including graphene, carbon nanotubes (CNTs), and carbon nanofibers, are particularly attractive because of their high electrical conductivity, chemical stability, and mechanical flexibility [74,75,76,77]. Graphene and reduced graphene oxide support rapid charge transport while remaining compatible with flexible substrates used in epidermal sensing devices [74]. Similarly, CNT networks provide conductive pathways that facilitate electron transfer between analyte molecules and electrode surfaces, enabling sensitive detection of metabolites such as urea and creatinine.

Two-dimensional transition-metal carbides and nitrides (MXenes) have recently emerged as promising materials for electrochemical biosensors [49,53,58,78]. Their metallic conductivity and hydrophilic surfaces support high loading of biomolecular recognition elements and efficient electron transfer. MXene-based electrodes have demonstrated low detection limits (0.2–10 nM depending on the analyte) and strong mechanical flexibility, making them attractive for wearable sensing platforms [53,78].

Noble metals such as gold and platinum provide stable conductive interfaces, while gold nanostructures enable thiol-based self-assembled monolayers for enzyme or antibody immobilization [79]. Metal nanostructures are also incorporated into electrode architectures to enhance catalytic activity and signal amplification [79,80,81]. Metal oxide nanostructures, such as zinc oxide, have similarly been explored due to their high surface area and electrochemical activity, which can improve sensitivity for detecting kidney-related biomarkers [82,83,84].

However, despite these advances, nanostructured electrodes primarily enhance analytical sensitivity under controlled conditions and do not fully address challenges associated with real-world deployment. In complex biofluids such as sweat and interstitial fluid, matrix effects, biofouling, and nonspecific adsorption can degrade signal quality and reduce long-term stability. Furthermore, improvements in detection limits do not necessarily translate to improved clinical relevance when biomarker concentrations in peripheral biofluids exhibit weak or variable correlation with systemic physiological states. While nanostructured materials are critical for advancing sensor performance, their impact is constrained by biointerface dynamics and biofluid variability.

### 4.2. Conductive Polymers and Hybrid Composites

Conductive polymers represent an important class of materials for wearable biosensors due to their mechanical flexibility, tunable electrical properties, and compatibility with soft substrates. Polymers such as polyaniline, polypyrrole, and poly(3,4-ethylenedioxythiophene) (PEDOT) are widely used as electrode coatings or sensing matrices, supporting efficient charge transport while maintaining mechanical stability under repeated deformation [48,59,85]. Molecularly imprinted polymers (MIPs) provide synthetic binding sites that mimic antibody selectivity while offering improved chemical stability compared to biological receptors [59,86]. These materials also provide porous structures that facilitate immobilization of enzymes or recognition elements for biomarker detection, while polymer matrices can help stabilize these components against mechanical stress and environmental degradation.

Hybrid composites combining conductive polymers with nanomaterials have demonstrated improved electrical performance and structural robustness [87,88]. Incorporation of graphene, carbon nanotubes (CNTs), or metal nanoparticles enhances conductivity and increases surface area for biomolecule immobilization, enabling flexible sensing interfaces that maintain electrochemical performance under mechanical strain. Additionally, conductive inks composed of these materials can be processed using scalable printing techniques, such as inkjet or screen printing, supporting large-scale fabrication of flexible biosensor electrodes [89,90].

However, despite these advantages, conductive polymer systems face limitations related to long-term stability, including polymer degradation, swelling, and interfacial delamination under continuous operation. While MIPs offer improved chemical robustness, they may exhibit lower binding affinity and slower kinetics compared to biological receptors, potentially limiting sensitivity and response time. Although conductive polymers and their composites are essential for enabling flexible and scalable wearable devices, their contribution to overall system performance remains constrained by broader challenges in biointerface stability, signal interpretation, and clinical translation.

### 4.3. Flexible Substrates and Stretchable Electronics

Flexible substrates such as polyimide, polyethylene terephthalate, thermoplastic polyurethane, and elastomers including polydimethylsiloxane are widely used to support wearable electronics due to their mechanical compliance and compatibility with microfabrication processes [44,91,92]. These materials enable conformal contact with the skin, facilitating stable interfacing between the sensing platform and the biological environment. Stretchable device architectures, including serpentine electrode geometries, microcracked conductive films, and elastomer-embedded conductive composites, have been developed to maintain electrical conductivity under repeated mechanical deformation. In addition, textile-based electronics represent an emerging platform in which conductive fibers and fabrics are integrated directly into garments, enabling distributed sensing networks that enhance user comfort and long-term wearability [60].

However, the mechanical flexibility of these systems introduces additional challenges related to device stability and signal reliability. Repeated deformation, mechanical fatigue, and interfacial delamination can degrade electrical performance over time, while strain-induced changes in electrode geometry may introduce signal variability. Furthermore, maintaining consistent skin contact under dynamic conditions remains a significant challenge, as motion artifacts and variations in contact pressure can affect signal quality and reproducibility.

### 4.4. Biointerface Engineering and Antifouling Strategies

Biointerface engineering therefore plays a critical role in maintaining reliable sensing performance in wearable systems. In biological environments, proteins, lipids, and salts can adsorb onto sensor surfaces, leading to biofouling that degrades analytical performance over time. These processes can block active sensing sites and increase interfacial resistance, reducing sensor sensitivity and stability during prolonged operation [53]. Antifouling coatings are commonly employed to mitigate these effects. Hydrophilic polymers such as polyethylene glycol and zwitterionic materials form hydrated surface layers that resist protein adsorption [61,62,63]. Hydrogel coatings are particularly attractive in wearable biosensors, as they can serve as both antifouling barriers and diffusion media that regulate analyte transport.

Biointerface engineering also improves the stabilization of biological recognition elements immobilized on sensor surfaces. Controlling enzyme or receptor orientation can enhance analyte recognition efficiency and extend operational stability. Therefore, conductive polymer matrices and hydrogel encapsulation are frequently used to protect biomolecules while maintaining electrochemical connectivity. In addition to enzyme-based sensing systems, enzyme-free recognition strategies (e.g., MIPs, aptamer-based sensors, and nanozyme catalysts) are increasingly explored to improve durability [61,62]. However, despite these advances, biofouling remains a persistent challenge in real-world wearable applications, particularly under continuous exposure to complex biofluids. Antifouling coatings can degrade over time, and strategies that limit nonspecific adsorption may also impede analyte diffusion, introducing trade-offs between selectivity and response time. Furthermore, while improved biointerfaces enhance short-term sensor performance, they do not fully resolve long-term stability issues or variability arising from biofluid composition and environmental conditions.

## 5. Challenges: Electrochemical Stability and Long-Term Sensor Performance

As shown in Table 3, significant variability exists in the correlation between wearable sensor measurements and blood biomarkers. Sensors targeting analytes such as urea typically rely on enzymatic electrochemical detection due to its high sensitivity and compatibility with miniaturized electronics. However, sustained operation in wearable environments introduces critical challenges, including enzyme degradation, pH fluctuations, biofouling, and reference electrode drift, all of which can compromise long-term measurement accuracy and reliability.

### 5.1. Enzyme Degradation and Catalytic Stability

Enzyme stability represents a major limitation of enzymatic electrochemical biosensors. Urease, commonly used for urea detection, catalyzes the hydrolysis of urea into ammonia and carbonic acid, generating ammonium and bicarbonate ions. Although highly efficient, urease activity is highly sensitive to environmental conditions, including temperature, hydration state, and pH [63,64,65,93]. Thermal stress can significantly reduce enzyme activity, with temperatures above approximately 40 °C causing irreversible denaturation, while exposure to 37–45 °C may reduce activity by 20–40% in unprotected enzyme films [65,93]. In wearable applications, these effects are exacerbated by fluctuating physiological and environmental conditions. Hydration variability during intermittent sweat secretion introduces repeated dehydration–rehydration cycles, which can destabilize immobilized enzymes and accelerate activity loss in thin-film systems [50,55]. Similarly, local pH variations resulting from biochemical reactions can alter enzyme kinetics, further contributing to signal drift over time.

Various strategies have been developed to improve enzyme stability, including hydrogel immobilization with osmolytes such as glycerol or trehalose, which can help preserve enzymatic activity [94,95]. Sol–gel encapsulation has also been shown to enhance stability; however, these approaches often introduce diffusion barriers that can reduce response speed and limit real-time sensing performance. While enzymatic sensing provides high analytical sensitivity, its long-term operational stability remains a critical bottleneck for continuous wearable monitoring. Enzyme degradation and environmental sensitivity can lead to signal drift and reduced reproducibility, limiting the reliability of these sensors for extended use. These challenges emphasize the need for alternative recognition strategies or hybrid approaches that combine enzymatic specificity with improved stability and calibration frameworks.

### 5.2. pH Drift and Buffering Artifacts

Urease typically exhibits optimal activity near pH 7.4–8.4, whereas sweat pH often ranges from 4.5 to 6.5 [32,50,55]. Urease-catalyzed hydrolysis increases the local pH near the electrode surface; however, in biofluids such as sweat, which have relatively low buffering capacity, these reactions can produce significant localized pH shifts. Exposure to acidic environments reduces enzyme stability and catalytic efficiency, further compromising sensor performance [32,51]. Because many electrochemical detection mechanisms are inherently pH-sensitive, these fluctuations can introduce calibration errors, signal drift, and reduced quantitative accuracy during continuous monitoring. In dynamic physiological conditions, such pH variability can decouple the measured signal from the true analyte concentration, limiting the reliability of real-time measurements.

To mitigate these effects, hydrogel matrices incorporating buffering agents such as phosphate or HEPES are commonly used in sensor architectures [51]. Properly optimized buffering systems can reduce pH fluctuations across clinically relevant urea concentrations. However, the incorporation of buffering layers may introduce additional diffusion barriers, potentially slowing sensor response and affecting temporal resolution. Therefore, pH regulation strategies involve trade-offs between stability and responsiveness, and do not fully eliminate variability in real-world wearable environments.

### 5.3. Biofouling and Surface Passivation

Despite advances in biointerface engineering, biofouling of electrode surfaces remains one of the most significant factors limiting the sustained analytical performance of wearable biosensors. Proteins, lipids, salts, and cellular debris in biofluids can accumulate on electrode surfaces during prolonged operation [57,62,96]. The adsorption of proteins and lipids leads to the formation of insulating layers, increasing interfacial impedance and restricting analyte diffusion to the sensing surface. These effects not only reduce sensor sensitivity but also contribute to signal drift and decreased reproducibility over time, particularly under continuous monitoring conditions. As a result, biofouling introduces time-dependent degradation that can compromise the reliability of long-term measurements in wearable systems.

Antifouling coatings are therefore widely employed to mitigate nonspecific adsorption by forming hydrated surface layers that resist protein adhesion. While such coatings can significantly reduce fouling compared with unmodified electrodes, their performance is often limited by gradual degradation and incomplete resistance under complex biological conditions. Moreover, increasing coating thickness to improve antifouling performance can introduce diffusion barriers that restrict analyte transport, leading to trade-offs between fouling resistance and sensor responsiveness. Moreover, biofouling remains a persistent challenge that cannot be fully eliminated through surface engineering alone, highlighting the need for complementary strategies such as periodic recalibration, self-cleaning interfaces, and data-driven correction methods to maintain long-term sensor performance.

### 5.4. Reference Electrode Stability

Stable reference electrode potentials are essential for accurate electrochemical measurements. Miniaturized Ag/AgCl electrodes are commonly used in wearable sensors due to their well-defined electrochemical potential and compatibility with flexible electronics. However, reference electrode stability can degrade in wearable environments due to chloride depletion, electrolyte exchange, and temperature fluctuations. As a result, unprotected reference electrodes may experience potential drift, which can translate into significant errors in measured analyte concentrations over time [54,97]. In continuous monitoring applications, even small potential shifts can accumulate, leading to progressive inaccuracies and reduced reproducibility. This effect is particularly problematic in low-concentration measurements, where minor voltage deviations can produce disproportionately large concentration errors.

Encapsulation strategies, including gelled electrolyte reservoirs and salt-bridge structures, have been developed to stabilize reference electrodes. Under controlled conditions, these designs can reduce drift to below approximately 1 mV/h [54]. However, maintaining such stability in dynamic wearable environments remains challenging, as evaporation, mechanical deformation, and biofluid exchange can compromise long-term performance. Reference electrode drift continues to represent a persistent source of measurement uncertainty in wearable electrochemical sensors. This necessitates the need for periodic recalibration, alternative reference designs, or correction algorithms to ensure reliable long-term operation.

### 5.5. System-Level Stabilization Strategies

Beyond materials-level solutions, system-level strategies are increasingly employed to enhance the long-term stability of wearable biosensors. Immobilization matrices such as polyacrylamide or alginate hydrogels provide hydration buffering and mechanical stabilization while maintaining analyte diffusion pathways. Crosslinking agents such as glutaraldehyde improve enzyme retention; however, excessive crosslinking can reduce catalytic turnover and limit sensor responsiveness. Permselective membranes such as Nafion can suppress interference from electroactive species while permitting diffusion of target analytes such as urea [18,96]. Additionally, environmental compensation strategies have been shown to improve measurement reliability [18]. For example, integrated temperature sensors enable correction for temperature-dependent enzymatic kinetics, reducing measurement error, while calibration protocols provide an additional layer of system-level stability [18]. Many wearable systems therefore adopt hybrid architectures that combine reusable electronics with disposable biochemical sensing modules to balance performance and longevity [16,20].

However, these mitigation strategies primarily address individual failure mechanisms and often introduce trade-offs between stability, sensitivity, and response time. For example, diffusion-limiting layers improve selectivity but may reduce temporal resolution, while stabilization strategies for biological components can compromise catalytic efficiency. As a result, no single engineering solution fully resolves the challenges associated with long-term operation in complex biological environments. The major technical barriers to long-term wearable CKD sensing and representative mitigation strategies are summarized in Table 5.

### 5.6. Comparative Analytical Performance Across Biofluids

Analytical sensitivity depends on both the sensing mechanism and the intrinsic analyte concentration within the sampled biofluid. Blood generally contains the highest and most stable concentrations of renal biomarkers, providing favorable analytical conditions [88,96]. In healthy adults, serum urea typically ranges from approximately 2.5–7.5 mM but may exceed 20 mM in advanced CKD [55]. Electrolytes such as potassium (5.0–6.0 mM) and sodium (135–150 mM) are tightly regulated [13,55]. Electrochemical sensors designed for blood measurements therefore achieve low detection limits (0.2–50 µM for urea) with wide dynamic ranges under controlled conditions [52].

In contrast, peripheral biofluids exhibit lower and more variable analyte concentrations. Sweat urea levels typically range from 5 to 50 mM but are strongly influenced by sweat rate and glandular activity [25,28,55,98], leading to higher detection limits (>100 µM) and reduced precision. ISF, which is in close biochemical equilibrium with plasma, provides improved correlation with blood biomarkers. Reported detection limits for ISF urea sensors range from 0.1 to 15 mM, with performance approaching within two- to three-fold of serum-based measurements [22].

However, temporal resolution differs significantly across biofluids. Blood-based measurements provide near-instantaneous readings, whereas ISF concentrations reflect plasma changes through diffusion across capillary endothelium, resulting in delays depending on tissue perfusion and sensor location [22,30,31,71]. Sweat-based sensing introduces even longer delays, as analytes must diffuse into eccrine glands and traverse glandular ducts before reaching the skin surface [98]. Although microfluidic sampling systems may reduce these delays by improving fluid transport, such approaches cannot fully eliminate transport-related lag.

These differences highlight a fundamental limitation of wearable biosensing: analytical performance is intrinsically constrained by the biofluid itself, including its analyte concentration, transport kinetics, and physiological variability. As a result, wearable measurements are often temporally and quantitatively decoupled from systemic biomarker levels, limiting their ability to provide direct clinical equivalence to blood-based diagnostics.

Recent advances in artificial intelligence and digital health platforms provide a promising pathway to address these challenges. Smartphone-connected electrochemical sensors and cloud-based systems enable continuous data acquisition, while machine learning models can be used to correlate wearable-derived signals with clinical biomarkers, compensate for temporal delays, and predict disease progression. These approaches suggest that the future of wearable CKD biosensing lies in the integration of sensing technologies with data-driven models capable of translating imperfect measurements into clinically actionable insights [14,16,44,45,46,47,48].

## 6. Clinical Translation and Regulatory Pathways

Translation of wearable biosensors to clinical practice remains constrained by validation, regulatory, and scalability requirements. As wearable biosensors evolve toward clinical decision-support tools, these translational considerations have become central to development.

### 6.1. Regulatory Frameworks

Prior to clinical deployment, wearable biosensors require comprehensive validation to demonstrate accuracy, precision, and reliability under physiologically relevant conditions [15,99]. For clinical acceptance, correlation coefficients exceeding 0.9 and error margins comparable to laboratory assays are typically required. However, achieving such performance in wearable systems is particularly challenging due to the inherent variability of peripheral biofluids, transport-related delays, and environmental fluctuations. As a result, wearable measurements often exhibit reduced correlation with blood-based standards, complicating validation against clinical reference methods. Because these devices operate on-body, validation must account for dynamic conditions including temperature fluctuations, perspiration, mechanical deformation, and variations in the skin–sensor interface. Accordingly, validation studies combine controlled laboratory experiments with human subject testing to assess real-world performance [99]. Evaluation of calibration stability, sensor drift, and resistance to biofouling is especially critical for continuous monitoring systems, where small errors can accumulate over time.

Wearable biosensors intended for medical use are regulated as medical devices. In the United States, the Food and Drug Administration governs such devices under the Federal Food, Drug, and Cosmetic Act, with many systems classified as Class II and cleared via the 510(k) pathway [100]. In Europe, devices are regulated under the Medical Device Regulation (MDR), which defines requirements for safety, clinical performance, and post-market surveillance [101]. Integration of digital analytics and machine learning introduces additional regulatory complexity. Systems incorporating diagnostic or predictive algorithms may be classified as Software as a Medical Device (SaMD) [102], requiring validation of algorithm performance, dataset integrity, and continuous monitoring for bias and drift.

Technology readiness level (TRL) frameworks provide a useful measure of system maturity [103]. Most wearable CKD biosensors remain at intermediate TRLs, corresponding to laboratory validation or early-stage human studies. Progression to higher TRLs is constrained not only by engineering challenges but also by the need to demonstrate consistent performance across diverse populations, where inter-individual variability in biofluid composition and physiology can significantly impact sensor outputs. Large-scale validation studies are therefore required to assess reliability, durability, user comfort, and clinical utility [104]. Demonstrating reproducible performance across cohorts is essential for regulatory approval and clinical adoption.

### 6.2. Commercialization and Scalability Challenges

Several barriers limit large-scale commercialization [103,104]. Manufacturing scalability remains a significant constraint, as nanomaterial-based and microfabricated components can be difficult to reproduce with consistent quality at industrial scale. Batch-to-batch variability in material properties may further contribute to inconsistencies in sensor performance, complicating quality control and regulatory approval. In addition, integration with healthcare infrastructure presents challenges, as continuous monitoring generates large volumes of data requiring secure storage, processing, and clinical interpretation. Compliance with data privacy regulations and cybersecurity requirements adds further complexity. Moreover, translating continuous data streams into clinically actionable insights remains an unresolved challenge, requiring robust algorithms capable of accounting for biofluid variability, temporal delays, and sensor drift.

### 6.3. Human Factors, User Experience, and Adoption Barriers

Patient adoption of wearable biosensors depends strongly on human-centered design. Factors such as device thickness, flexibility, skin compatibility, breathability, and ease of use directly influence wearability and long-term adherence [105]. However, achieving consistent user compliance remains a significant challenge, particularly in continuous monitoring applications. Skin irritation, device detachment, and excessive bulk can limit sustained use, especially among elderly patients who may exhibit increased skin sensitivity and reduced dexterity. While thin, flexible materials and breathable adhesive interfaces can improve comfort, maintaining reliable skin–sensor contact under real-world conditions remains difficult, often introducing variability in signal quality. Simplified operation and minimal maintenance are essential, yet many current systems still require calibration, replacement, or user intervention, which can reduce usability across diverse patient populations.

Another major barrier to clinical adoption is the interpretation of continuous data streams [106]. Wearable systems generate complex, time-resolved datasets that must be translated into clinically meaningful outputs. However, variability in biofluid composition, sensor drift, and temporal delays can complicate the relationship between measured signals and true physiological states, limiting clinician confidence in these systems. Without robust interpretation frameworks, clinicians may be reluctant to rely on wearable-derived data for decision-making. Accordingly, the development of reliable algorithms capable of converting sensor data into actionable clinical indicators is critical. Data visualization tools, predictive analytics, and defined intervention thresholds can facilitate interpretation and integration into clinical workflows [106]. Nevertheless, the effectiveness of these approaches depends on the availability of high-quality, validated datasets and the ability to account for inter-individual variability and environmental influences.

Equally important is standardized validation demonstrating consistent correlation with established clinical biomarkers across diverse populations. Educational initiatives may improve familiarity among healthcare providers; however, widespread adoption will ultimately depend on demonstrating that wearable systems can provide reliable, interpretable, and clinically actionable information comparable to conventional diagnostics. As a result, human-centered design and data interpretation challenges are not merely usability concerns but fundamental barriers to clinical translation, reinforcing the need for integrated solutions that combine robust sensing, adaptive calibration, and data-driven modeling.

## 7. Future Perspectives

The transition from individual biosensors to integrated digital health systems represents a critical step toward clinical implementation of wearable technologies for CKD monitoring. Early wearable sensors primarily targeted single analytes, whereas modern platforms increasingly combine multiple sensing modalities with wireless communication and cloud-based analytics [15,16]. Advances in flexible electronics, low-power microprocessors, and wireless networking have enabled wearable devices capable of simultaneously monitoring biochemical and physiological signals [17,18]. These integrated systems support continuous biomarker tracking, real-time data transmission, and remote patient monitoring. Such capabilities form the foundation of digital nephrology, an emerging paradigm that integrates biosensing technologies with computational analytics to enable proactive and personalized kidney disease management [107].

Continuous wearable monitoring generates large datasets requiring advanced computational analysis. Sensor fusion techniques combine heterogeneous data streams from multiple sensors to extract clinically meaningful insights. Artificial intelligence (AI) and machine learning (ML) algorithms are increasingly applied to wearable biosensor datasets to enable predictive healthcare applications [106,108]. These algorithms can model patient-specific physiological baselines and detect deviations that may indicate early deterioration in kidney function. By analyzing longitudinal datasets, AI-driven platforms may ultimately enable early detection of electrolyte imbalances, prediction of dialysis complications, and identification of rapid declines in renal function before clinical symptoms appear.

However, the effectiveness of AI-driven analytics is fundamentally dependent on the quality and reliability of input data. Variability in biofluid composition, sensor drift, and transport-related delays can introduce noise and bias into wearable datasets, potentially limiting model accuracy and generalizability. Further, inconsistent calibration and inter-individual physiological differences complicate the development of robust predictive models across diverse patient populations.

Remote monitoring offers the potential for earlier detection of complications such as hyperkalemia, fluid overload, or declining renal function. Integration of wearable biosensors with digital communication infrastructure enables remote monitoring ecosystems capable of transforming CKD management. In these systems, wearable devices transmit physiological data to smartphones or gateway devices, which relay information to cloud-based platforms for storage, analysis, and clinical review. However, the translation of continuous sensor data into clinically actionable alerts remains challenging, as defining reliable intervention thresholds requires large-scale, longitudinal validation across heterogeneous patient populations. 

This end-to-end digital workflow enables continuous CKD monitoring using wearable biosensors. Biochemical (e.g., creatinine, urea, cystatin C, electrolytes) and physiological signals are acquired through wearable sensing platforms and transmitted via secure wireless communication protocols (e.g., Bluetooth Low Energy, NFC, Wi-Fi, or cellular networks) to mobile and cloud-based infrastructures. Data management layers perform preprocessing, quality control, and anonymization, followed by advanced analytics using artificial intelligence and machine learning models for feature extraction, multimodal data fusion, trend analysis, and risk prediction.

Processed outputs, including biomarker trends, risk scores, and alert notifications, are integrated into clinician-facing dashboards to support risk stratification, decision-making, and timely intervention. Interoperability with healthcare systems (e.g., electronic health records and telemedicine platforms) enables seamless clinical integration. Cross-cutting enablers such as data security, privacy compliance (e.g., HIPAA/GDPR), interoperability standards (e.g., FHIR/HL7), and patient engagement support robust system deployment. A closed-loop feedback mechanism facilitates continuous learning, model updating, and personalized care, ultimately improving early detection, treatment outcomes, and quality of life in CKD patients.

**Figure 4:**
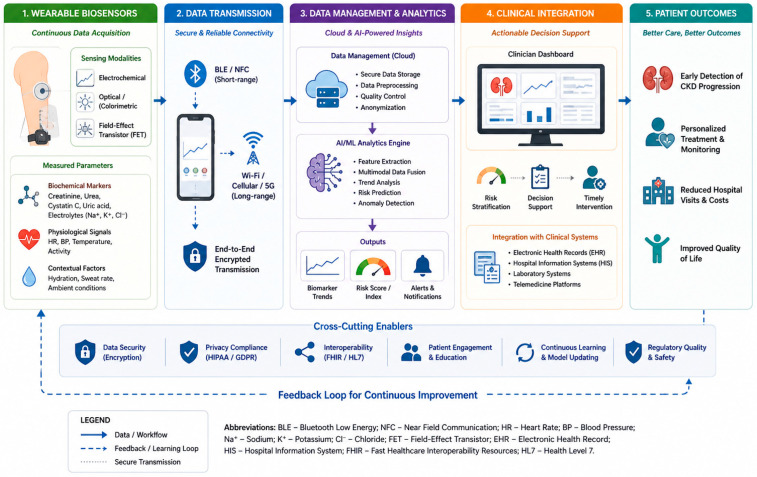
End-to-end framework for wearable CKD monitoring, illustrating continuous biosensing, secure data transmission, AI-driven analytics, clinical decision support, and improved patient outcomes, supported by cross-cutting enablers such as data security, interoperability, and regulatory compliance.

At the system level, integration with electronic health record systems may enhance the clinical utility of wearable monitoring platforms by incorporating biosensor data into existing healthcare workflows. In such digital nephrology ecosystems, wearable devices function as distributed sensing nodes while centralized analytics platforms generate clinically actionable insights [107]. Building on these integrated digital frameworks, wearable biosensors are poised to transform CKD diagnosis and management by enabling continuous monitoring of biochemical and physiological parameters. Concurrently, progress in wireless communication and cloud-based analytics has enabled integration into digital health ecosystems for remote monitoring and data-driven clinical decision-making [44,109]. While such end-to-end digital workflows demonstrate significant promise, their clinical utility is contingent upon robust validation, data standardization, and seamless integration with existing healthcare systems, which remain ongoing challenges. A conceptual digital nephrology monitoring ecosystem integrating wearable sensing, mobile devices, and clinical decision-support platforms is illustrated in Figure 4.

From a translational perspective, regulatory frameworks must evolve to accommodate continuous-monitoring systems integrating hardware, software, and AI-driven analytics. Pathways addressing SaMD and adaptive algorithms are critical for the deployment of wearable digital health platforms. Recent advances further highlight this potential. Skin-conformal sensor arrays now enable real-time, multiplexed analysis of sweat biomarkers, while multisensing patches integrating electrochemical and physical sensors (e.g., pH, temperature) improve accuracy and contextual interpretation. Ion-selective electrochemical platforms have also enabled real-time monitoring of sodium and potassium during activity, supporting sweat-based assessment of electrolyte balance.

Despite these advances, key barriers to clinical adoption remain. Large-scale longitudinal studies are required to validate correlations between wearable sensor outputs and established clinical biomarkers, enabling the definition of clinically actionable thresholds. Moreover, the integration of hardware, software, and adaptive AI models introduces additional complexity in regulatory approval, data governance, and clinical trust. Although digital nephrology frameworks represent a transformative direction for CKD management, their success will depend on the convergence of reliable sensing technologies, standardized validation protocols, and robust data-driven interpretation models capable of translating continuous, imperfect measurements into clinically meaningful insights.

## 8. Positioning of This Review Within Current Literature

Recent reviews have significantly advanced the field of wearable biosensors, particularly in materials innovation, device architectures, and biofluid-based sensing (Table 6). For instance, recent studies have explored electrochemical sensing platforms and flexible device integration for real-time biomarker monitoring [110], while others have emphasized 2D material-enabled systems and graphene-based technologies with enhanced sensitivity and flexibility [111,112]. Parallel efforts have focused on microfluidic-enabled wearable sensing and autonomous biofluid handling [113], as well as system-level approaches incorporating AI-driven data processing and multimodal sensing [114].

Despite these advances, the literature remains fragmented. Most reviews either (i) focus on materials and device engineering without disease-specific context [111,112], (ii) emphasize clinical deployment without sufficient consideration of sensing mechanisms, or (iii) discuss wearable platforms without linking biomarker transport dynamics to underlying disease pathophysiology [114]. Even recent CKD-focused studies primarily address biomarker identification or monitoring strategies without integrating wearable technologies, materials design, and translational readiness within a unified framework.

This review addresses these gaps by establishing an integrated materials-to-clinic framework that incorporates renal pathophysiology and biomarker relevance in CKD, biofluid transport mechanisms (sweat and ISF), advanced sensing materials and nanostructures, energy systems and self-powered architectures, and translational considerations including validation, regulatory pathways, and technology readiness levels. By bridging these traditionally disconnected domains, this work provides a systems-level perspective linking fundamental sensing mechanisms to clinical applicability.

This approach not only clarifies key limitations across the field but also defines design and validation priorities for advancing wearable CKD biosensors toward clinical deployment. As such, this review serves as both a comprehensive synthesis of current knowledge and a roadmap for next-generation, clinically deployable systems. Importantly, future progress will depend on integrating sensing technologies with data-driven models capable of translating continuous, biofluid-derived signals into clinically actionable insights.

## 9. Conclusions

Wearable biosensors are emerging as promising technologies for improving the diagnosis and management of chronic kidney disease by enabling continuous monitoring of key biomarkers. Advances in flexible electronics, nanostructured sensing materials, microfluidic sampling systems, and minimally invasive interfaces have expanded the capability of wearable devices to monitor metabolites and electrolytes in peripheral biofluids such as sweat and interstitial fluid. However, significant challenges remain before these systems can achieve widespread clinical adoption. Key limitations include biofouling, sensor drift, enzyme instability, and variability in biofluid transport dynamics, all of which can affect long-term analytical reliability. Addressing these challenges will require continued progress in biointerface engineering, stable recognition elements such as molecularly imprinted polymers and aptamer-based systems, and integrated multimodal sensing architectures. In parallel, advances in digital health platforms, data analytics, and regulatory frameworks will be essential for translating wearable CKD biosensors from laboratory prototypes to clinically deployable monitoring systems capable of enabling personalized and proactive nephrology care. This review uniquely integrates biofluid transport physics with wearable sensor design and clinical translation, providing a framework for next-generation CKD monitoring systems.

## Figures and Tables

**Table 1:** A summary of key CKD biomarkers and their relevance to wearable biosensing.

Biomarker	Primary Biofluid(s)	Physiological Role/Clinical Significance	Typical Physiological Range	Wearable Sensing Feasibility	Refs.
Creatinine	Blood, Interstitial fluid, Sweat	Indicator of renal filtration efficiency; used to estimate glomerular filtration rate (eGFR)	~0.6–1.3 mg/dL (serum)	Moderate—requires sensitive detection due to low sweat concentrations	[18,22,30,31]
Urea	Blood, Sweat, ISF	Major nitrogenous waste product of protein metabolism; accumulates during renal impairment	~7–20 mg/dL (serum)	High—relatively abundant and detectable in sweat	[25,32,33]
Uric Acid	Blood, Sweat, Saliva	End product of purine metabolism; elevated levels associated with CKD progression and cardiovascular risk	~3.5–7.2 mg/dL (serum)	High—detectable in multiple peripheral biofluids	[34,35]
Electrolytes (Na^+^, K^+^)	Sweat, ISF, Blood	Regulate fluid balance, nerve conduction, and cellular homeostasis; electrolyte imbalance is common in CKD	Na^+^ ~135–145 mM, K^+^ ~3.5–5.0 mM	High—compatible with ion-selective wearable sensors	[22,25,36]
Albumin	Urine	Marker of glomerular barrier damage; early indicator of kidney injury	ACR ≥ 30 mg/g indicates albuminuria	Low–Moderate—sampling requires urine collection	[37,38]
Cystatin C	Blood	Alternative biomarker for estimating GFR; less affected by muscle mass than creatinine	~0.6–1.3 mg/L	Low—primarily blood-based measurement	[14,16]

**Table 2:** Comparison of wearable sensing modalities relevant to CKD biomarker monitoring.

Modality	Target	Transduction Principle	Strengths	Limitations	Refs.
Electrochemical	Urea, K^+^, creatinine	Electrochemical signal (current/voltage)	High sensitivity, compact systems	Biofouling and signal drift	[13,22,25,36]
FET-based	Electrolytes, toxins	Semiconductor channel modulation	Label-free detection	Debye screening in ionic fluids	[39,40]
Optical	Urea, proteins	Absorbance or fluorescence	Multiplexing capability	Ambient light interference	[34,35]
Bioimpedance	Fluid status	Tissue impedance measurement	Noninvasive volume estimation	Motion artifacts	[40,41,42,43]

**Table 3:** Performance metrics of representative wearable biosensors for CKD-relevant biomarkers.

Biofluid	Biomarker	Sensing Modality	Detection Limit	Linear Range	Response Time	Correlation with Blood	Key Refs.
Sweat	Urea	Electrochemical	~µM	µM–mM	Seconds–minutes	Moderate (variable)	[49,50,51,52]
Sweat	Na^+^/K^+^	Electrochemical (ISE)	µM	mM range	Seconds	Good for trends	[25,36,53,54]
Sweat	Uric acid	Electrochemical	µM	µM–mM	Seconds–minutes	Limited validation	[18,22,55]
ISF	Glucose	Electrochemical/microneedle	µM	µM–mM	Seconds	High (clinically validated)	[30,31,56]
ISF	Urea	Electrochemical	µM	µM–mM	Minutes	Moderate–high	[30,31,54]
ISF	Electrolytes	Electrochemical	µM	mM range	Seconds–minutes	High	[16,23,57,58,59,60,61,62,63,64,65,66]
Sweat	Urea	Optical	µM	µM–mM	Minutes	Semi-quantitative	[34,35]
Tissue	Fluid status	Bioimpedance	N/A	N/A	Continuous	Clinically correlated	[41,42,43]

**Table 4:** Comparison of biofluids for wearable CKD monitoring.

Biofluid	Access	Correlation	Advantages	Limitations	Clinical Relevance	Refs.
Blood	Invasive	Direct	Gold standard	Not continuous	High	[14,16,18]
ISF	Microneedles	High	Accurate	Time lag	High	[18,22,30,31]
Sweat	Epidermal	Moderate	Noninvasive	Variability	Moderate	[18,22,25,30,31,36,68]
Saliva/Tears	Noninvasive	Low	Easy sampling	Weak CKD link	Limited	[37,38,69,70]

**Table 5:** Key engineering challenges in wearable CKD biosensors and representative mitigation strategies.

Challenge	Mechanism	Impact	Engineering Solution	Key References
Biofouling	Protein/lipid adsorption	Increased impedance (>50%)	PEG/zwitterionic coatings	[42]
Enzyme degradation	Thermal/pH instability	Decreased activity (20–50%)	Hydrogel encapsulation	[63]
pH drift	Urease byproducts	ΔpH (0.5–1.5 units)	Buffered hydrogels	[32,33]
Reference drift	Cl^−^ depletion	10–30 mV/day	Salt-bridge encapsulation	[36]
ISF lag	Diffusion delay	Temporal offset	Predictive modeling	[30,31]

**Table 6:** Comparative analysis of recent wearable biosensor reviews and identification of research gaps in CKD monitoring.

Study	Year	Focus Area	Biofluid Scope	CKD Focus	Materials Depth	System Integration	Clinical Translation	Key Gap
[33]	2024	CKD wearable monitoring	Sweat, ISF	Moderate	Moderate	Moderate	Limited	No materials– power integration
[110]	2025	Clinical wearable biosensors (pediatrics)	Multi-biofluid	None	Low	Moderate	Strong	Lacks materials and sensing depth
[111]	2025	Electrochemical wearable biosensors	Multi-biofluid	None	High	Moderate	Limited	No clinical/disease integration
[112]	2025	Microfluidic wearable biosensing	Sweat, ISF	None	Moderate	High	Moderate	Weak biomarker–disease linkage
[113]	2025	2D-material wearable systems	Multi-biofluid	None	High	High	Limited	No CKD-specific mapping
[114]	2025	Graphene wearable biosensors	Multi-biofluid	None	High	Moderate	Limited	No translational framework
[115]	2025	Comprehensive wearable biosensing systems	Multi-biofluid	None	Moderate	High	Moderate	No disease-specific synthesis
[116]	2024	CKD biomarkers	Blood, urine	Strong	Low	None	Moderate	No wearable systems
[117]	2024	Electrochemical + mechanical wearables	Multi-biofluid	None	Moderate	Moderate	Limited	No disease-specific translation
This Work	2026	CKD + materials + biofluid transport + TRL	Sweat + ISF + systemic mapping	Strong	High	High	Strong	Addresses current gaps

## Data Availability

No new data were created or analyzed in this study.
